# Effects of COVID-19 on in-hospital cardiac arrest: incidence, causes, and outcome – a retrospective cohort study

**DOI:** 10.1186/s13049-021-00846-w

**Published:** 2021-02-08

**Authors:** Kevin Roedl, Gerold Söffker, Dominik Fischer, Jakob Müller, Dirk Westermann, Malte Issleib, Stefan Kluge, Dominik Jarczak

**Affiliations:** 1 Department of Intensive Care Medicine, University Medical Centre Hamburg-Eppendorf, Martinistraße 52, 20246 Hamburg, Germany; 2 Department of Anaesthesiology, University Medical Centre Hamburg-Eppendorf, Hamburg, Germany; 3 Department of Anaesthesia, Tabea Hospital, Hamburg, Germany; 4 Department of Interventional and General Cardiology, University Heart Centre Hamburg, Hamburg, Germany

**Keywords:** COVID-19, Corona virus disease, Multiple organ failure, Intensive care unit, SARS-COV-2, Cardiac arrest, Cardiopulmonary resuscitation, In-hospital cardiac arrest

## Abstract

**Background:**

Severe acute respiratory syndrome coronavirus-2 (SARS-CoV-2), an emerging virus, has caused a global pandemic. Coronavirus disease 2019 (COVID-19), caused by SARS-CoV-2, has led to high hospitalization rates worldwide. Little is known about the occurrence of in-hospital cardiac arrest (IHCA) and high mortality rates have been proposed. The aim of this study was to investigate the incidence, characteristics and outcome of IHCA during the pandemic in comparison to an earlier period.

**Methods:**

This was a retrospective analysis of data prospectively recorded during 3-month-periods 2019 and 2020 at the University Medical Centre Hamburg-Eppendorf (Germany). All consecutive adult patients with IHCA were included. Clinical parameters, neurological outcomes and organ failure/support were assessed.

**Results:**

During the study period hospital admissions declined from 18,262 (2019) to 13,994 (2020) (− 23%). The IHCA incidence increased from 4.6 (2019: 84 IHCA cases) to 6.6 (2020: 93 IHCA cases)/1000 hospital admissions. Median stay before IHCA was 4 (1–9) days. Demographic characteristics were comparable in both periods. IHCA location shifted towards the ICU (56% vs 37%, *p* < 0.01); shockable rhythm (VT/VF) (18% vs 29%, *p* = 0.05) and defibrillation were more frequent in the pandemic period (20% vs 35%, *p* < 0.05). Resuscitation times, rates of ROSC and post-CA characteristics were comparable in both periods. The severity of illness (SAPS II/SOFA), frequency of mechanical ventilation and frequency of vasopressor therapy after IHCA were higher during the 2020 period. Overall, 43 patients (12 with & 31 without COVID-19), presented with respiratory failure at the time of IHCA. The Horowitz index and resuscitation time were significantly lower in patients with COVID-19 (each *p* < 0.01). Favourable outcomes were observed in 42 and 10% of patients with and without COVID-19-related respiratory failure, respectively.

**Conclusion:**

Hospital admissions declined during the pandemic, but a higher incidence of IHCA was observed. IHCA in patients with COVID-19 was a common finding. Compared to patients with non-COVID-19-related respiratory failure, the outcome was improved.

**Supplementary Information:**

The online version contains supplementary material available at 10.1186/s13049-021-00846-w.

## Background

Originating from Wuhan, China, a series of pneumonias of initially unknown cause emerged in December 2019 [1, 2]. A novel coronavirus (severe acute respiratory syndrome coronavirus 2 (SARS-CoV-2)) spread and caused a pandemic [3, 4]. Although many patients have a mild course of disease, a considerable number of patients suffer from severe illness with rapid progression to acute respiratory distress syndrome (ARDS) or/and end-organ failure [1–3].

COVID-19 has resulted in high rates of hospitalization and a high number of patients requiring intensive care unit (ICU) treatment [5, 6]. The course of disease can be complicated, and can potentially lead to cardiac arrest (CA) for several reasons, as shown by various studies [7–10]. An increase in out-of-hospital cardiac arrest (OHCA) cases was observed during the COVID-19 pandemic [11, 12]. However, little is known about the CA risk in hospitalized patients with COVID-19 [10, 13–15]. Poor in-hospital survival following in-hospital cardiac arrest (IHCA) in patients with COVID-19 has been described, and mortality ranged from 88 to 100% [9, 10, 13, 15]. However, data on in-hospital cardiac arrest (IHCA) in patients with respiratory failure with and without COVID-19 are scarce.

In general, an estimated 290,000 adults suffer from IHCA in the United States annually [16, 17]. IHCA is often unexpected and presents as an acute event; every hospitalized patient can potentially be affected. Different studies have shown abnormal vital signs as predictors of IHCA [18, 19]. Therefore, rapid response teams and the use of warning scores have been established [20]. Although most IHCAs occur in general wards [21, 22], a considerable number of IHCAs occur in the ICU [23]. The incidence of IHCA varies greatly in the literature (1–5/1000 hospital admissions) [16, 17]. Rates of survival to hospital discharge range from 13 to 22% [24].

However, data on IHCA during the COVID-19 pandemic are very limited. In the present study we aimed to investigate the occurrence, determinants, outcome and post-CA course of patients suffering from IHCA during the COVID-19 pandemic and before.

## Methods

### Study population, design and ethics

This was a retrospective analysis of data prospectively recorded at the University Medical Centre Hamburg-Eppendorf (Germany). All consecutive adult patients suffering an IHCA during a 3-month period in 2019 and 2020 were included. The following time periods were compared: 2019 (February 27–May 28) and 2020 (February 27–May 27). For post-CA care all patients were treated at the Department of Intensive Care Medicine, which cares for all critically ill adult patients of the hospital and includes 12 ICUs (total capacity: 142 ICU beds). The study complied with the Declaration of Helsinki. The Ethics Committee of the Hamburg Chamber of Physicians was informed about the study (No.: WF-152/20). The requirement for informed patient consent was waived due to the use of only anonymized data collected during routine clinical care. The last day of follow-up was September 30, 2020.

### Inclusion and exclusion criteria

We included all consecutive adult patients (≥ 18 years) with an IHCA event. Patients < 18 years of age and patients or with a prior OHCA event and/or re-arrest after hospital admission were not considered as an incident IHCA and were therefore excluded.

### Study definitions and patient management

IHCA was defined as cessation of circulation, and therefore, an indication for chest compression and/or cardiac defibrillation in patients who had a pulse and circulation at the time of hospital admission. Sustained return of spontaneous circulation (ROSC) was defined as stable circulation for at least 20 min. Assessment of neurological outcome was performed within routine clinical practice using cerebral performance categories (CPCs) after the IHCA and during follow-up. A CPC score of 1–2 was defined as a favourable neurological outcome, and a score of 3–5 was defined as an unfavourable neurological outcome. Survival was assessed through the end of the ICU stay. Cardiopulmonary resuscitation and post-CA care were performed in accordance with the European Resuscitation Council guidelines [25]. Data were collected according to Utstein-style guidelines [26]. Cardiac failure was defined as the need for inotrope/vasopressors (dobutamine, epi−/norepinephrine) during the first 72 h after CA [27]. Hypoxic liver injury (HLI) was diagnosed according to established criteria [28]. COVID-19 was defined as a positive result on a reverse transcriptase-polymerase chain reaction, and only laboratory-confirmed cases were counted as COVID-19. ARDS was defined using the PaO_2_/FiO_2_ ratio (Horowitz index) according to the Berlin definition [29–31]. The severity of illness was evaluated by the sequential organ failure assessment (SOFA) score [32] and simplified acute physiology (SAPS II) [33] score. The Charlson comorbidity index (CCI) [34] was calculated in all patients.

### Data collection

Data were collected through electronic patient data management systems (PDMS, Integrated Care Manager® (ICM), Version 9.1 – Draeger Medical, Luebeck, Germany; Soarian Clinicals, Version 4.3.200 – Cerner Health Service, Inc.) and consisted of age, sex, comorbidities, admission diagnosis, length of ICU-stay, treatment modalities, organ support (mechanical ventilation, vasopressor, renal replacement therapy (RRT), blood transfusions, antibiotics, antivirals, etc.), laboratory parameters and further clinical parameters of interest through the end of ICU-stay. Pre-existing medication was recorded based on known regular medications and medication on admission. Laboratory assessment was performed daily as part of the clinical routine.

### Statistical analysis

The results are presented as counts and relative frequencies or medians and 25–75% interquartile ranges (IQRs). Binary variables were compared via chi-square analysis/Fisher’s exact test, as appropriate. Metric variables were compared via the Mann-Whitney U test. We used multivariable Cox regression to investigate factors associated with mortality and unfavourable outcomes. Factors of clinical relevance were selected and included. A *p*-value < 0.05 was considered statistically significant. Statistical analysis was conducted using IBM SPSS Statistics Version 24.0 (IBM Corp., Armonk, NY). The study was prepared in accordance with the STROBE (STrengthening the Reporting of OBservational studies in Epidemiology) recommendations.

## Results

### Study population

**Fig. 1:**
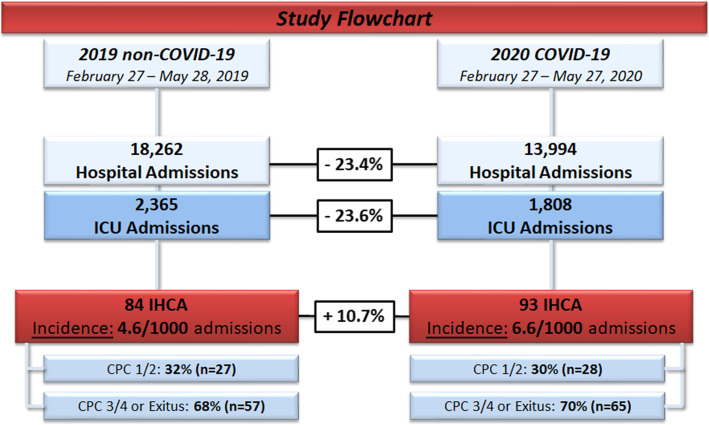
Study flow chart

During the two study time periods, namely 2019-non-COVID-19 (February 27–May 28) and 2020-COVID-19 (February 27–May 27), a total of 18,262 and 13,994 inpatients were treated at the University Medical Centre Hamburg-Eppendorf, respectively. We identified 84 (2019-non-COVID-19 period) and 93 (2020-COVID-19 period) patients suffering from IHCA during the two study periods; these patients were included in the present study (see Study Flow-Chart Fig. 1).

### Baseline and cardiac arrest characteristics of the study population

Detailed baseline and IHCA characteristics are shown in Tables 1 and 2. Patients were predominantly male (68%, *n* = 120); the median age was 70 (57–78) years. In this study, comorbidities were frequent, and a median CCI of 3 (2–5) was observed. Arterial hypertension (67%, *n* = 118) was the leading comorbidity. Furthermore, common comorbidities were history of malignant condition (tumour, haematologic malignancy) (33%, *n* = 58), coronary heart disease (35%, *n* = 62), diabetes mellitus type II (21%, *n* = 38), chronic respiratory disease (20%, *n* = 36) and chronic kidney disease (18%, *n* = 32). The reasons for hospital admission were medical in 74% (*n* = 131) of the patients, unplanned surgery in 14% (*n* = 24) and planned surgical in 13% (*n* = 22). The median duration from hospital admission to IHCA was 4 (1–9) days. The IHCA location was non-ICU in 53% (*n* = 94). The initial cardiac rhythm was shockable (VT/VF) in 24% (*n* = 42); defibrillation during CPR was performed in 28% (*n* = 50). The median total resuscitation time was 5 (2–17) minutes. Sustained ROSC was observed in 80% (*n* = 142), and cardiac re-arrest was observed in 30% (*n* = 53). A mechanical chest compression system was used in 11% (*n* = 19). Aetiology of the IHCA was presumed cardiac in 37% (*n* = 66). Due to refractory IHCA 5% (n = 9) received extracorporeal-CPR (E-CPR).

**Table 1:** Baseline characteristics of patients with in-hospital cardiac arrest stratified according the 2019 (Non-COVID-19) and 2020 (COVID-19) period

*Parameters*	*All patients* *(n = 177)*	*2019 – Non-COVID-19 Period* *(n = 84)*	*2020 – COVID-19 Period* *(n = 93)*	*p-value*
Demographics				
Age, years *median (IQR)*	70 (57–78)	72 (57–78)	68 (57–78)	0.721
Sex, male *n (%)*	120 (68)	60 (71)	60 (65)	0.206
Height, cm *median (IQR)*	172 (165–180)	175 (168–180)	170 (165–179)	0.109
Weight, kg *median (IQR)*	76 (65–85)	73 (65–84)	78 (67–86)	0.207
BMI, kg/m^2^ *median (IQR)*	25 (23–29)	25 (23–27)	26 (24–29)	0.077
Comorbidities				
Charlson comorbidity index, pts.; *median (IQR)*	3 (2–5)	3 (2–5)	3 (2–5)	0.802
Arterial hypertension, *n (%)*	118 (67)	49 (58)	69 (74)	**0.019**
Coronary heart disease, *n (%)*	66 (37)	29 (35)	33 (35)	0.510
Chronic kidney disease, *n (%)*	32 (18)	13 (15)	19 (20)	0.255
Chronic respiratory disease, *n (%)*	36 (20)	20 (24)	16 (17)	0.183
Diabetes, *n (%)*	38 (21)	20 (24)	18 (19)	0.312
Malignant condition, *n (%)*	58 (33)	23 (27)	35 (38)	0.098
COVID-19				
Confirmed COVID-19, *n (%)*	12 (7)	–	12 (13)	
Positive test to ICU, days *median (IQR)*	10 (3–17)	–	10 (3–17)	
Positive test to IHCA, days *median (IQR)*	17 (14–28)	–	17 (14–28)	
Cough, *n (%)*	–	–	7 (58)^a^	
Shortness of breath, *n (%)*	–	–	3 (25)^a^	
Fever, *n (%)*	–	–	6 (50)^a^	
Fatigue, *n (%)*	–	–	3 (25)^a^	
Myalgia, *n (%)*	–	–	1 (8)^a^	
Reason of hospital admission				
*Surgical*				
planned, *n (%)*	22 (13)	7 (8)	15 (16)	0.089
unplanned, *n (%)*	24 (14)	14 (17)	10 (11)	0.177
Medical*, n (%)*	131 (74)	63 (75)	68 (73)	0.455
Characteristics – before CA				
Heart rate /min; *median (IQR)*	91 (77–111)	91 (80–108)	91 (75–111)	0.922
MAP mmHg; *median (IQR)*	70 (62–82)	72 (66–82)	70 (60–82)	0.549
Outcome				
Overall mortality, *n (%)*	95 (54)	41 (49)	54 (58)	0.140
Discharged from ICU alive, *n (%)*	82 (46)	43 (51)	39 (42)	0.140
Length of stay – ICU, days *median (IQR)*	5 (2–17)	6 (2–17)	5 (2–16)	0.592

*Abbreviations:* *cm* Centimeter; *BMI* Body mass index; *kg* Kilogram; *ICU* Intensive care unit; *IQR* Interquartile range; *n* Number; *pts*. Points; *min* Minute; *MAP* Mean arterial pressure; *COVID-19* Coronavirus disease 2019; *IHCA* In-hospital cardiac arrest; ^a^ in relation to positive tested patients (*n* = 12)

**Table 2:** Cardiac arrest and ICU-characteristics of the study cohort stratified in the 2019 (No-COVID-19) and 2020 (COVID-19) period

*Parameters*	*All patients* *(n = 177)*	*2019 – No-COVID-19* *(n = 84)*	*2020 – COVID-19* *(n = 93)*	*p-value*
Cardiac arrest Characteristics				
*Location of IHCA*				**0.009**
ICU, *n (%)*	83 (47)	31 (37)	52 (56)	
Non-ICU, *n (%)*	94 (53)	53 (63)	41 (44)	
Initial rhythm - shockable (VT/VF), *n (%)*	42 (24)	15 (18)	27 (29)	0.058
Defibrillation, *n (%)*	50 (28)	17 (20)	33 (35)	**0.018**
Sustained ROSC, *n (%)*	142 (80)	65 (77)	77 (83)	0.237
Cardiac re-arrest, *n (%)*	53 (30)	27 (32)	26 (30)	0.329
Presumed cardiac cause, *n (%)*	66 (37)	35 (42)	31 (33)	0.161
Epinephrine – total dose, mg, *median (IQR)*	2 (1–4)	2 (1–4)	2 (1–4)	0.978
*Resuscitation time, min; median (IQR)*				
No-flow	0 (0–0)	0 (0–0)	0 (0–0)	0.300
Total resuscitation time	5 (2–17)	4 (1.5–14)	5 (2–20)	0.204
Targeted temperature management, *n (%)*	56 (32)	27 (32)	29 (31)	0.509
Use of mechanical compression system, *n (%)*	19 (11)	12 (14)	7 (8)	0.114
E-CPR, *n (%)*	9 (5)	5 (6)	4 (4)	0.436
ICU – Characteristics				
*Severity of illness*				
SAPS II (pts.) *median (IQR)*	45 (35–55)	44 (35–56)	47 (35–54)	0.837
SOFA – after CA (pts.) *median (IQR)*	12 (9–14)	11 (8–13)	12 (10–14)	0.060
SOFA – 24 h after CA (pts.) *median (IQR)*	11 (7–14)	11 (7–14)	11 (8–14)	0.923
*Physiological parameters – post CA*				
Heart rate – after CA *median (IQR)*	96 (77–115)	94 (77–110)	96 (77–125)	0.232
MAP – after CA *median (IQR)*	73 (63–88)	76 (62–92)	72 (63–82)	0.324
*Lab values – post CA median (IQR)*				
Lactate – highest after CA, mmol/l	4.5 (1.9–9.1)	4.8 (2–9.1)	4.4 (1.9–8.2)	0.965
pH – lowest after CA	7.26 (7.07–7.36)	7.26 (7.07–7.34)	7.26 (7.10–7.36)	0.755
*Procedures/Complications – post CA*				
Mechanical ventilation, *n (%)*	124 (70)	55 (65)	69 (74)	**0.031**
Vasopressor therapy, *n (%)*	120 (68)	49 (58)	71 (76)	**0.025**
Renal replacement therapy, *n (%)*	26 (15)	11 (13)	15 (16)	0.243
Coronary angiography, *n (%)*	21 (12)	14 (17)	7 (8)	**0.050**
Hypoxic liver injury, *n (%)*	35 (20)	16 (19)	19 (20)	0.484
Cholestasis – bilirubin > 2 mg/dl, *n (%)*	45 (25)	22 (26)	23 (25)	0.480

*Abbreviations: CA* Cardiac arrest; *E-CPR* Extracorporeal cardiopulmonary resuscitation; *ICU* Intensive care unit; *IQR* Inter quartile range; *n* Number; *min* Minute; *mg* Milligram; *mmol/l* Millimole per liter; *pts*. Points; *ROSC* Return of spontaneous circulation; *SAPS* Simplified Acute Physiology Score; *SOFA* Sequential Organ Failure Assessment; *VF* Ventricular Fibrillation; *VT* Ventricular Tachycardia; *MAP* Mean arterial pressure; *COVID-19* Coronavirus disease 2019; *IHCA* In-hospital cardiac arrest

### Differences during the pandemic period and before

Tables 1 and 2 show a comparison of the detailed baseline and IHCA characteristics comparing between the study periods. During the 2020-COVID-19 period hospital admissions and ICU admissions declined from 18,262 to 13,994 (− 23%) and from 2365 to 1808 (− 24%), respectively. The incidence of IHCA increased from 4.6 to 6.6/1000 hospital admissions. Demographic characteristics (age, sex and BMI) and comorbidities (as measured by CCI) were comparable between the groups. Arterial hypertension was significantly more common in patients during the COVID-19 period (2019 Non-COVID-19 period: 58% vs 2020 COVID-19 period: 74%). The most common reason for hospital admission was medical care, which did not differ between the time periods. The IHCA location was primarily non-ICU during 2019-non-COVID-19 period and primarily in the ICU during the 2020-COVID-19 period (*p* < 0.01). A shockable rhythm (18% vs 29%) was more frequently observed during the COVID-19 period, and the use of defibrillation (20% vs 35%) was significantly higher. The rates of sustained ROSC (77% vs 83%), cardiac re-arrest (32% vs 30%) and total epinephrine use (2 mg vs 2 mg) were comparable in both study periods. The median resuscitation time was 4 min vs. 5 min and did not differ significantly between the groups. Mechanical compression systems were used more frequently during the 2019 period (14% vs 8%). Targeted temperature management post-CA was used in 32% of patients in the whole cohort, and the frequency was similar in both study periods. The SAPS II and SOFA score post-CA were higher during the 2020-COVID-19 period. During the ICU stay mechanical ventilation was performed more frequently during the 2020-COVID-19 period (65% vs 74%, *p* < 0.05). Vasopressor therapy was more commonly used during the 2020-COVID-19 period (58% vs 76%). Liver dysfunction was frequent during both study periods; 20% suffered from hypoxic liver injury and 25% suffered from cholestasis.

### IHCA and COVID-19

During the aforementioned 2020 time period, 144 patients with COVID-19 were treated as inpatients at our centre. Of these, 75 patients were treated in the normal ward, and 69 patients were critically ill and therefore treated in the ICU. Twelve patients (10%) with COVID-19 treated at our hospital suffered from IHCA. All patients had severe respiratory failure either due to pneumonia or due to the development of ARDS. The median times from the first positive SARS-CoV-2 test to the ICU and to IHCA were 10 (3–17) days and 17 (14–28) days, respectively. The most common symptoms of COVID-19 were cough (*n* = 7; 58%), fever (*n* = 6; 50%), shortness of breath and fatigue (*n* = 3 for each, 25%). None of the IHCAs occurred outside the ICU. All patients had a primary non-shockable rhythm (PEA/Asystole) and ROSC. The median resuscitation time was1.5 (0.5–3.5) minutes. For detailed characteristics of patients with COVID-19, see Tables 1, 2, 3 and Supplementary Tables 1 and 2.

**Table 3:** Cardiac arrest characteristics of patients with severe respiratory failure with and without COVID-19

*Parameters*	*All patients* *(n = 43)*	*Severe respiratory failure no-COVID-19* *(n = 31)*	*Severe respiratory failure – COVID-19* *(n = 12)*	*p-value*
Demographics				
Age, years *median (IQR)*	65 (50–75)	65 (50–77)	65 (56–74)	0.565
Sex, male, *n (%)*	34 (79)	25 (81)	9 (75)	0.471
BMI, kg/m^2^ *median (IQR)*	27 (24–31)	26 (24–30)	28 (26–33)	0.314
Charlson comorbidity index, pts. *median (IQR)*	3 (1.5–6)	4 (2.5–6)	2 (1–2)	**0.003**
Characteristics of respiratory failure				
Respiratory support (before CA)				
Non-invasive ventilation *n (%)*	7 (16)	3 (10)	4 (33)	0.125
Mechanical ventilation *n (%)*	22 (51)	17 (55)	5 (42)	0.148
Cause of respiratory failure (at CA)				
Pneumonia *n (%)*	37 (86)	25 (81)	12 (100)	**0.001**
ARDS *n (%)*	24 (56)	12 (39)	10 (83)	**0.000**
Horowitz index (PaO_2_/FiO_2_-ratio)				
Worst Horowitz index, mmHg, *median (IQR)*	84 (57–148)	90 (57–149)	82 (59–107)	0.503
Horowitz index after CA, mmHg, *median (IQR)*	97 (76–145)	101 (78–152)	89 (69–19)	**0.007**
ARDS Management				
Prone Positioning *n (%)*	8 (19)	2 (6)	6 (50)	0.437
Neuromuscular Blockage *n (%)*	6 (14)	1 (3)	5 (42)	0.306
Corticosteroids *n (%)*	11 (26)	4 (13)	7 (58)	0.563
Inhaled Vasodilators *n (%)*	9 (21)	3 (10)	6 (50)	0.437
Cardiac arrest Characteristics				
*Location*				**0.009**
ICU, *n (%)*	37 (86)	25 (81)	12 (100)	
Non-ICU, *n (%)*	6 (14)	6 (19)	0 (0)	
Initial Rhythm - Shockable (VT/VF), *n (%)*	6 (14)	6 (19)	0 (0)	0.255
Sustained ROSC, *n (%)*	40 (93)	28 (90)	12 (100)	0.364
Epinephrine – total dose, mg, *median (IQR)*	1 (1–2)	2 (1–2.5)	1 (1–1.3)	0.310
*Resuscitation time, min; median (IQR)*				
No-Flow	0 (0–0)	0 (0–0)	0 (0–0)	1
Total resuscitation time	4 (1.8–8.5)	5 (2–10)	1.5 (0.5–3.5)	**0.008**
Targeted temperature management, *n (%)*	10 (23)	8 (26)	2 (17)	0.339
E-CPR, *n (%)*	0 (0)	0 (0)	0 (0)	1
ICU – Characteristics				
*Severity of illness*				
SAPS II (pts.) *median (IQR)*	44 (36–52)	42 (35–49)	50 (40–56)	0.485
SOFA – after CA (pts.) *median (IQR)*	14 (12–16)	14 (12–17)	15 (13–16)	0.202
SOFA – 24 h after CA (pts.) *median (IQR)*	13 (11–16)	13 (11–15)	14 (10–16)	0.145
*Lab values – post CA median (IQR)*				
Lactate – highest after CA, mmol/l	4.6 (1.6–8.5)	4.8 (1.5–10)	4.2 (3.1–4.8)	0.765
pH – lowest after CA	7.21 (7.15–7.32)	7.22 (7.06–7.32)	7.2 (7.19–7.3)	0.889
*Procedures/Complications – post CA*				
Vasopressor therapy, *n (%)*	40 (93)	29 (94)	11 (92)	0.505
Renal replacement therapy, *n (%)*	22 (51)	12 (39)	10 (83)	**0.009**
Coronary angiography, *n (%)*	0 (0)	0 (0)	0 (0)	1
Hypoxic liver injury, *n (%)*	11 (26)	7 (23)	4 (33)	0.201
Cholestasis – bilirubin > 2 mg/dl, *n (%)*	15 (58)	10 (32)	5 (42)	0.190

*Abbreviations: CA* Cardiac arrest; *cm* Centimeter; *E-CPR* Extracorporeal cardiopulmonary resuscitation; *ICU* Intensive care unit; *IQR* Inter quartile range; *kg* Kilogram; *n* Number; *min* Minute; *mg* Milligram; *mmol/l* Millimole per liter; *pts*. Points; *ROSC* Return of spontaneous circulation; *SAPS* Simplified Acute Physiology Score; *SOFA* Sequential Organ Failure Assessment; *VF* Ventricular Fibrillation; *VT* Ventricular Tachycardia; *MAP* Mean arterial pressure; *COVID-19* Coronavirus disease 2019; *IHCA* In-hospital cardiac arrest; *BMI* Body mass index

### Characteristics of IHCA in patients with or without COVID-19 related severe respiratory failure

Detailed characteristics are shown in Table 3 and Supplementary Table 1 and 2. Overall, 25% (*n* = 43) of patients had severe respiratory failure at the time of IHCA and were selected. Of those 28% (*n* = 12) suffered from COVID-19 pneumonia. Demographic characteristics (age, sex, BMI) were comparable between patients with severe respiratory failure who did not have COVID-19. Comorbidities, represented by the CCI were significantly lower (4 vs 2 points; *p* < 0.01) in patients with non-COVID-19 related severe respiratory failure. In total, 68% received non-invasive or invasive mechanical ventilation prior to IHCA. Overall, 56% (*n* = 24) of patients suffered from ARDS at the time of IHCA and ARDS was more frequently observed in patients with COVID-19. In addition, the Horowitz index after IHCA was significantly lower in patients with COVID-19. ARDS management, including prone positioning, neuromuscular blockage, corticosteroids and inhaled vasodilatory treatment, was comparable in both groups. IHCA within the ICU was significantly more frequent in patients with COVID-19 than in those without COVID-19-related severe respiratory failure. The most common initial rhythm was non-shockable in both groups. The use of epinephrine was comparable in both groups. The total resuscitation time was longer in patients with non-COVID-19 related severe respiratory failure (median 5 vs 1.5 min; *p* < 0.01). The severity of illness at ICU admission and after IHCA was comparable between the groups. During the ICU stay, RRT was more frequent (*p* < 0.01) in patients with COVID-19. Laboratory values before and after IHCA were comparable between the groups. Furthermore, physiological parameters before and after IHCA did not differ significantly.

### Survival and functional outcome

Of the 177 included patients who had an IHCA event, 99 (54%) did not survive the ICU-stay. Fifty-six patients (32%) died within 24 h after the IHCA. At ICU discharge 31% (*n* = 55) had favourable neurological outcomes (CPC I/II). Rates did not differ significantly between the two study periods (2019: 32% - 2020: 30%). In patients with COVID-19, the rates of favourable neurological outcomes (CPC I/II) were higher than those in patients with non-COVID-19-related severe respiratory failure (42% vs 10%). Cox regression analysis revealed that the SOFA-score after IHCA [HR 1.17, 95% CI (1.00–1.36); *p* < 0.05], CCI [HR 1.13, 95% CI (1.01–1.26); *p* < 0.05] and low-flow time [HR 1.07, 95% CI (1.01–1.12); *p* < 0.05] were significantly associated with unfavourable neurological outcome or ICU-mortality within patients with severe respiratory failure (see Supplementary Table 3).

## Discussion

In this study investigating the effects of the COVID-19 pandemic on IHCA, we found that the incidence of IHCA was increased, the location of IHCA shifted towards the ICU and CA-characteristics were changed. To our knowledge, this is the first study evaluating the effect of the COVID-19 pandemic on IHCA. Furthermore, this is the first study comparing the IHCA characteristics of patients suffering from severe respiratory failure that was and was not related to COVID-19 at time the of IHCA.

The COVID-19 pandemic led to a higher incidence of OHCA and worse short-term outcomes [11, 12]. Different mechanisms suggesting direct effects of COVID-19 and effects from lockdown were proposed [35]. However, to date, no data on how the pandemic has affected IHCA exist. Due to the rapid spread and surge of patients with COVID-19, elective admissions to hospitals were cancelled to create more capacity for patients suffering from COVID-19. This was impressively demonstrated by a 23% decrease in hospital admissions during the COVID-19 period. Although hospital admissions decreased substantially, an 11% increased incidence of IHCA was observed. The reported incidence of IHCA in the literature is 1–5/1000 hospital admissions; during the pandemic period, the incidence of 6.6/1000 hospital admissions exceeded reported rates [16, 17]. Different factors could explain this finding. First, patients with COVID-19 are at high risk of IHCA due to rapidly worsening respiratory failure eventually leading to IHCA if not promptly treated. Of interest, explainable deterioration of SpO_2_ and high FiO_2_, but only minor abnormalities in other vital signs, as well as higher early warning scores, have recently been described as predictors for outcome [36, 37]. Second, the severity of illness at ICU admission was substantially higher than that in the non-COVID-19 period. Although an early ICU admission strategy was followed, this may be explained by delayed or disrupted contact with the healthcare system due to lockdown measures, leading to delayed hospital admission in general.

Furthermore, we observed substantial differences according to IHCA characteristics during the study periods. During the pandemic period, the IHCA location shifted more towards the ICU, which may be explained by earlier ICU admission of deteriorating patients. Moreover, the rate of shockable rhythm and defibrillation increased and we observed high rates of ROSC. These observed differences are potentially explained by higher rates of IHCA occurring in the ICU and a faster response to deterioration due to higher nurse/doctor staffing. Interestingly, the duration of resuscitation was slightly longer during the pandemic period. CPR, an aerosol-generating procedure, exposes healthcare workers to a risk of viral transmission. Therefore, the use of personal-protective equipment is of central importance but could have contributed to the delayed initiation of CPR. Furthermore, the lower rate of presumed cardiac aetiology is important. However, resuscitation times were shorter than those in previous studies [23].

Overall, one quarter of patients presented with an initial shockable rhythm, which is in line with previous studies and can be explained by the low rate of cardiac aetiology of the IHCA. However, half of the patients were in the ICU before IHCA and suffered from high severity of illness, and the rate of MV and vasopressor support was associated with a non-shockable rhythm [38, 39].

The occurrence of IHCA among hospitalized patients with COVID-19 commonly ranges from 6 to 14% [9, 10]. We confirmed these results and found an incidence of 8%. The outcome after IHCA in patients with COVID-19 is worse, and high mortality rates, ranging from 88 to 100%, have been reported [9, 10, 13, 15, 40]. These reports led to a controversial discussion about futility and appropriateness of care in patients suffering from COVID-19. However, in our small cohort, we observed a distinctly lower mortality than previously reported, although we observed comparable IHCA characteristics, including similar rates of non-shockable initial rhythm, resuscitation time and occurrence of IHCA in the ICU. The lower mortality in our cohort can be a consequence of several reasons. First, a considerably lower number of patients were on MV or RRT before CA, demonstrating a lower severity of illness. Moreover, we followed a strategy of early admission to the ICU in patients with COVID-19 for closer monitoring and early initiation of supportive care. This could also correspond to the high rate of ROSC observed in our cohort and is probably related to continuous monitoring and higher nurse/doctor staffing. Second, earlier reports originated from regions with an excessive case load which potentially led to overwhelmed healthcare systems playing an important role in appropriate patient care [13, 15]. However, decisions on futility and withholding CPR are difficult and must be based on a multifactorial approach that takes the severity of illness, current organ support and the patient’s directive into account.

**Fig. 2:**
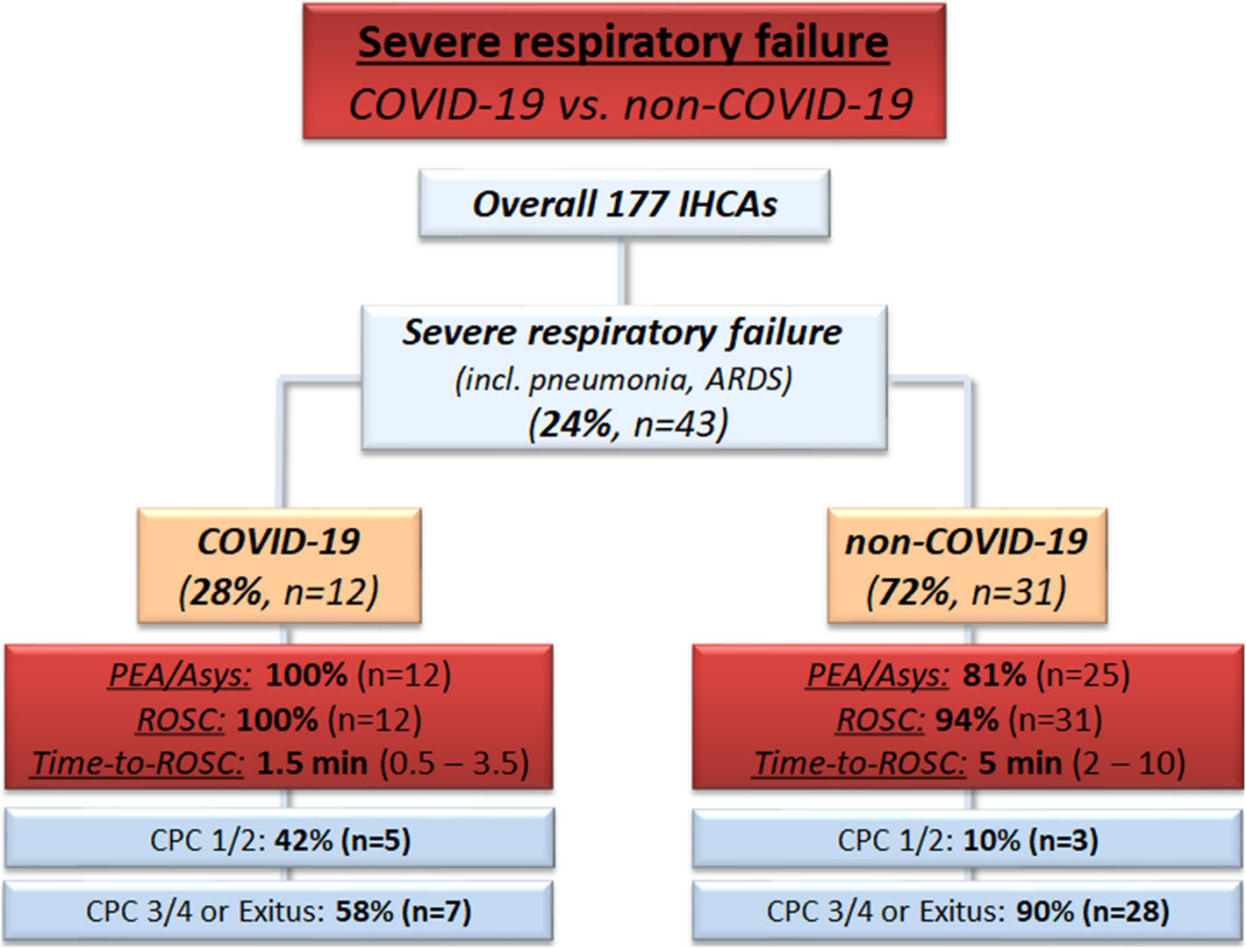
Outcome of patients with severe respiratory failure – stratified to COIVD-19 and non-COVID-19

SARS-CoV-2 primarily affects the respiratory system which can lead to rapid deterioration and severe respiratory failure. Recent clinical studies reported high mortality following IHCA events in patients with COVID-19 [9, 10, 13]. As patients with COVID-19 primarily suffer from respiratory failure, comparing the characteristics and outcomes of IHCA to patients suffering from respiratory failure related and not related to COVID-19 seems reasonable. However, this is the first study comparing patients with severe respiratory failure not related to COVID-19 at the time of IHCA with patients suffering from COVID-19. We observed that patients with COVID-19 had a lower comorbidity rate and substantially lower Horowitz index before and after IHCA. In patients suffering from ARDS, we observed comparable therapeutic approaches. Furthermore, a high rate of IHCA occurring in the ICU was observed, and correspondingly, a substantially lower resuscitation time was observed. In our cohort, we observed that IHCA often occurred during tracheal intubation. This may be a consequence of delayed decisions for tracheal intubation. However, this should lead to higher awareness of the timing of intubation in patients with progredient respiratory failure. A higher number of patients with COVID-19 required RRT and had liver injury (HLI/cholestasis) contributing to the higher severity of illness after IHCA. However, direct viral effects cannot be entirely excluded. Moreover, CCI, SOFA scores and resuscitation time were identified as mortality predictors in these patients. Of interest, a substantially higher number of patients with COVID-19 had a favourable outcome compared to other patients with severe respiratory failure (Fig. 2). Larger future studies must confirm these results and their implications on outcome.

This study has several limitations. First, our study included a small number of patients. Larger cohorts are needed to confirm our findings. Second, the data were derived from a single centre and were collected retrospectively. However, the data were documented prospectively in the PDMS by trained ICU staff. Third, we show the results of an experienced high-volume CA centre. Thus, the results might not generally be transferable to other, less experienced, settings. Fourth, the study was conducted early during the pandemic. Changes in clinical practice, due to more experience with COVID-19, could have changed affecting the incidence and outcome of IHCA especially in critically ill patients.

## Conclusions

In conclusion, this is the first study evaluating IHCA occurrence and outcomes during the COVID-19 pandemic in comparison to recent years. Hospital admissions declined during the pandemic, but a higher incidence of IHCA was observed, which could be attributed to multifactorial influences and must be further evaluated. Approximately 10% of hospitalized patients with COVID-19 suffered from IHCA, and outcomes were improved compared with those previously reported and comparable to those of patients with other aetiologies of respiratory failure not related to COVID-19.

## Supplementary Information

**Additional file 1: Supplementary Table 1.** Pre-existing comorbidities of patients with severe respiratory failure at time of cardiac arrest stratified according patients with and without COVID-19. **Supplementary Table 2.** Characteristics of patients with severe respiratory failure before and after cardiac arrest stratified according with and without COVID-19. **Supplementary Table 3.** Cox regression model for factors associated with ICU-mortality and unfavorable neurological outcome (CPC III/IV) of patients with IHCA and severe respiratory failure.

## Data Availability

The datasets supporting the conclusions of this article are included within the article.

## Abbreviations

ARDS: Acute respiratory distress syndrome
Asys: Asystole
BMI: Body mass index
CA: Cardiac arrest
CCI: Charlson comorbidity index
COVID-19: Coronavirus disease 2019
CPC: Cerebral performance categories
E-CPR: Extracorporeal cardiopulmonary resuscitation
HLI: Hypoxic liver injury
ICU: Intensive care unit
IHCA: In-hospital cardiac arrest
IQR: Interquartile range
MV: Mechanical ventilation
OHCA: Out-of-hospital cardiac arrest
PEA: Pulseless electrical activity
ROSC: Return of spontaneous circulation
RRT: Renal replacement therapy
SAPS: Simplified acute physiology score
SARS-CoV-2: Severe acute respiratory syndrome coronavirus 2
SOFA: Sequential organ failure assessment score
VT: Ventricular tachycardia
VF: Ventricular fibrillation
