# Supplementary material for: Effects of COVID-19 on in-hospital cardiac arrest: incidence, causes, and outcome – a retrospective cohort study
Source: Scand J Trauma Resusc Emerg Med. 2021 Feb 8;29:30. doi: 10.1186/s13049-021-00846-w (PMC7868866; doi:10.1186/s13049-021-00846-w)
Supplement: Supplementary file 1 — Additional file 1: Supplementary Table 1. Pre-existing comorbidities of patients with severe respiratory failure at time of cardiac arrest stratified according patients with and without COVID-19. Supplementary Table 2. Characteristics of patients with severe respiratory failure before and after cardiac arrest stratified according with and without COVID-19. Supplementary Table 3. Cox regression model for factors associated with ICU-mortality and unfavorable neurological outcome (CPC III/IV) of patients with IHCA and severe respiratory failure. [file 13049_2021_846_MOESM1_ESM.docx]

Supplementary Table 1 – Pre-existing comorbidities of patients with severe respiratory failure at time of cardiac arrest stratified according patients with and without COVID-19

| *Parameters* | *All patients*  *(n=43)* | *Severe respiratory failure no-COVID-19*  *(n=31)* | *Severe respiratory failure COVID-19*  *(n=12)* | *p-value* |
| --- | --- | --- | --- | --- |
| AIDS *n (%)* | 2 (5) | 2 (6) | 0 (0) | 0.515 |
| Cerebral arterial disease *n (%)* | 8 (19) | 7 (23) | 1 (8) | 0.356 |
| Chronic lung disease *n (%)* | 10 (23) | 9 (29) | 1 (8) | 0.149 |
| Chronic kidney disease *n (%)* | 6 (14) | 5 (16) | 1 (8) | 0.455 |
| Congestive heart disease *n (%)* | 9 (21) | 9 (29) | 0 (0) | **0.023** |
| Connective tissue disease *n (%)* | 4 (9) | 4 (13) | 0 (0) | 0.255 |
| Coronary heart disease *n (%)* | 13 (30) | 11 (35) | 2 (17) | 0.339 |
| Dementia *n (%)* | 0 (0) | 0 (0) | 0 (0) | 1 |
| Diabetes mellitus *n (%)* | 13 (30) | 9 (29) | 4 (33) | 0.356 |
| Liver cirrhosis *n (%)* | 5 (12) | 5 (16) | 0 (0) | 0.296 |
| Peripheral arterial disease *n (%)* | 6 (14) | 6 (19) | 0 (0) | 0.121 |
| Malignancy (Tumour) *n (%)* | 15 (35) | 10 (32) | 5 (42) | 0.404 |

*Abbreviations:* AIDS, acquired immune deficiency syndrome; n, number;

Supplementary Table 2 - Characteristics of patients with severe respiratory failure before and after cardiac arrest stratified according with and without COVID-19

| *Parameters* | *All patients*  *(n=43)* | *Severe respiratory failure no-COVID-19*  *(n=31)* | *Severe respiratory failure COVID-19*  *(n=12)* | *p-value* |
| --- | --- | --- | --- | --- |
| Pre-/Post-Arrest – Characteristics  *Lab values median (IQR)*  Bilirubin – before CA (mg/dl)  Bilirubin – after CA (mg/dl)  Bilirubin – before CA (mg/dl)  Aspartate-aminotransferase – before CA (U/l)  Aspartate-aminotransferase – after CA (U/l)  Aspartate-aminotransferase – 24h after CA (U/l)  Alanin-aminotransferase – before CA (U/l)  Alanin-aminotransferase – after CA (U/l)  Alanin-aminotransferase – 24h after CA (U/l)  Creatinine – before CA (mg/l)  Creatinine – after CA (mg/l)  Creatinine – 24h after CA (mg/l)  C-reactive protein – before CA (mg/l)  C-reactive protein – after CA (mg/l)  C-reactive protein – 24h after CA (mg/l)  Procalcitonin – before CA (qg/l)  Procalcitonin – after CA (qg/l)  Procalcitonin – 24h after CA (qg/l)  Thrombocytes – before CA (G/l)  Thrombocytes – after CA (G/l)  Thrombocytes – 24h after CA (G/l)  Troponin – before CA (pg/nl)  Troponin – after CA (pg/nl)  Troponin – 24h after CA (pg/nl) | 0.6 (0.5 – 1)  0.7 (0.4 – 1.2)  0.7 (0.4 – 1.35)  55 (36 – 89)  85 (40 – 295)  93 (54 – 270)  43 (22 – 66)  59 (31 – 163)  68 (35 – 133)  1.12 (0.79 – 1.87)  1.39 (1.07 – 1.99)  1.06 (0.94 – 1.63)  131 (72 – 249)  113 (58 – 171)  119 (71 – 175)  0.98 (0.36 – 1.82)  2.07 (0.88 – 3.8)  1.83 (1.21 – 3.93)  195 (30 – 311)  209 (140 – 253)  191 (129 – 243)  40 (10 – 142)  95 (69 – 185)  143 (94 – 230) | 0.6 (0.4 – 1.7)  0.55 (0.4 – 1.3)  0.7 (0.3 – 1.4)  52 (31 – 90)  73 (34 – 277)  111 (59 – 310)  43 (24 – 85)  49 (28 – 158)  90 (34 – 171)  1.14 (0.83 – 1.9)  1.39 (1.2 – 2)  1 (0.90 – 1.21)  130 (65 – 218)  118 (41 – 177)  150 (89 – 177)  0.94 (0.36 – 5.68)  3.8 (1.24 – 5.74)  5.09 (2.08 – 76.7)  186 (49 – 301)  233 (171 – 259)  206 (146 – 251)  28 (10 – 84)  144 (69 – 207)  338 (124 – 357) | 0.7 (0.5 – 1)  0.8 (0.6 – 0.8)  0.7 (0.5 – 1.2)  71 (53 – 89)  109 (77 – 451)  89 (54 – 105)  55 (19 – 56)  83 (47 – 193)  61 (51 – 83)  0.91 (0.77 – 1.77)  1.5 (1.05 – 1.96)  1.52 (0.97 – 1.71)  148 (89 – 284)  87 (85 – 154)  88 (71 – 128)  1.11 (0.56 – 1.65)  1.09 (0.89 – 2.18)  1.26 (1.07 – 1.98)  195 (18 – 371)  184 (113 – 213)  165 (54 – 219)  71 (14 – 169)  93 (65 – 165)  119 (82 – 223) | 0.549  0.151  0.121  0.495  0.214  0.497  0.815  0.427  0.522  0.349  0.334  0.191  0.397  0.238  0.941  0.808  0.440  0.077  0.764  0.061  0.175  0.630  0.541  0.833 |
| *ICU Intervention - in place before CA**  Mechanical ventilation *n (%)*  Non-invasive mechanical ventilation *n (%)*  Renal replacement therapy *n (%)*  Vasopressor therapy *n (%)* | 22 (51)  7 (33)  8 (19)  31 (72) | 17 (55)  3 (10)  5 (16)  22 (71) | 5 (42)  4 (33)  3 (25)  9 (75) | 0.148  0.125  0.659  0.592 |
| *Physiological parameters - before CA*  Heart rate *n (%)*  Mean arterial pressure *n (%)*  *Physiological parameters – 24h after CA*  Heart rate *n (%)*  Mean arterial pressure *n (%)* | 101 (90 – 116)  80 (61 – 80)  82 (73 – 100)  70 (61 – 79) | 100 (88 – 111)  70 (64 – 82)  80 (73 – 98)  71 (62 – 81) | 105 (96 – 136)  70 (61 – 77)  83 (74 – 113)  68 (64 – 75) | 0.340  0.908  0.521  0.550 |
| *Antihypertensive medication history*  Angiotensin-converting enzyme inhibitor *n (%)*  Angiotension-receptor blocker *n (%)* | 12 (28)  6 (14) | 7 (23)  5 (16) | 5 (42)  1 (8) | 0.485  0.569 |

*Abbreviations:* CA, cardiac arrest; g/dl, gram per deciliter; G/l, giga per liter; h, hours; ICU, intensive care unit; IQR, inter quartile range; n, number; mg/l, milligram per liter; mmol/l, millimole per liter; pg/nl, picogram per nanoliter; *only available for patients staying in ICU before CA

Supplementary Table 3 – Cox regression model for factors associated with ICU-mortality and unfavorable neurological outcome (CPC III/IV) of patients with IHCA and severe respiratory failure

| Covariables | HR (95% CI) | *p* value |
| --- | --- | --- |
| Total resuscitation time (min) | 1.066 (1.012 – 1.124) | **0.017** |
| Charlson comorbidity index (pts.) | 1.126 (1.005 – 1.262) | **0.042** |
| Non-shockable rhythm (PEA/Asys) (yes vs no) | 1.019 (0.316 – 3.986) | 0.872 |
| Horowitz index post-CA (mmHg) | 1.001 (0.999 – 1.003) | 0.426 |
| SOFA after CA (pts.) | 1.167 (1.004 – 1.355) | **0.044** |

***Abbreviations:*** CA, cardiac arrest; CI confidence interval; HR, multivariable adjusted hazard ratio; min, minute; SOFA**,** Sequential Organ Failure Assessment; pts, points; PEA, pulseless electrical activity; Asys, Asystole;
